# Patent foramen ovale-associated stroke repeatedly misdiagnosed as cerebral small vessel disease: A case report

**DOI:** 10.1097/MD.0000000000032996

**Published:** 2023-03-17

**Authors:** Yiwen Zhang, Ruihan Zhu, Zhenzhen Yu, Songqi Hou, Jianping Niu

**Affiliations:** a Neurology Department, The Second Affiliated Hospital of Xiamen Medical College, Xiamen, China; b Imaging Department, The Second Affiliated Hospital of Xiamen Medical College, Xiamen, China.

**Keywords:** case report, contrast-enhanced transcranial Doppler, patent foramen ovale, screening, stroke

## Abstract

Transthoracic echocardiography (TTE) is the most commonly used screening method for cardiac structural abnormalities. However, it may lead to a missed diagnosis of partial patent foramen ovale (PFO)-associated stroke.

**Case presentation:**

A 60-year-old male was admitted to the hospital for recurrent left limb weakness with or without slurred speech for 14 months. No stroke-related cardiac structural abnormality was detected during repeated TTE, and the patient was diagnosed with cerebral small vessel disease. Finally, right-to-left shunt was detected by contrast-enhanced transcranial Doppler. Subsequently, the patient was diagnosed with PFO-associated stroke by transesophageal echocardiography and contrast transesophageal echocardiography.

**Conclusions:**

TTE has a low detection rate of PFO, such that it is easily missed. Contrast-enhanced transcranial Doppler is easy to operate and should be promoted as a supplementary measure to stroke etiological investigation and primary PFO screening.

## 1. Introduction

Patent foramen ovale (PFO) refers to the permanent fissure-like channel resulting from the non-fully fused primary and secondary septum in children >3-years-old. The sensitivity of transthoracic echocardiography (TTE) is low due to the anatomy of PFO.^[1]^ Presently, transesophageal echocardiography (TEE) is the first choice in the diagnosis of PFO.^[2]^ Moreover, PFO provides a potential channel for right-to-left shunt, which leads to paradoxical embolism.^[3]^ A previous study has shown that PFO is the most common cause of stroke for unknown reasons.^[4]^ As a persistent anatomical abnormality, the presence of other stroke risk factors does not affect the pathogenesis of PFO. However, TTE is the most commonly used screening method for cardiac structural abnormalities. Further examinations, such as TEE and contrast-enhanced transcranial Doppler (c-TCD), were performed only in the absence of other risk factors or for unknown causes of stroke. This might lead to a missed diagnosis of stroke caused by PFO.^[5]^ A case of patent foramen ovale-associated stroke (PFO-AS) has been reported in this study. The patient presented repeated minor strokes and was repeatedly misdiagnosed with cerebral small vessel disease. This case report could be used for peer reference.

## 2. Case presentation

A 60-year-old male patient was admitted to The Second Affiliated Hospital of Xiamen Medical College, Xiamen, China, in January 2022.

*Chief complaint*: Recurrent left limb weakness with or without slurred speech for 14 months.

**Figure 1.**
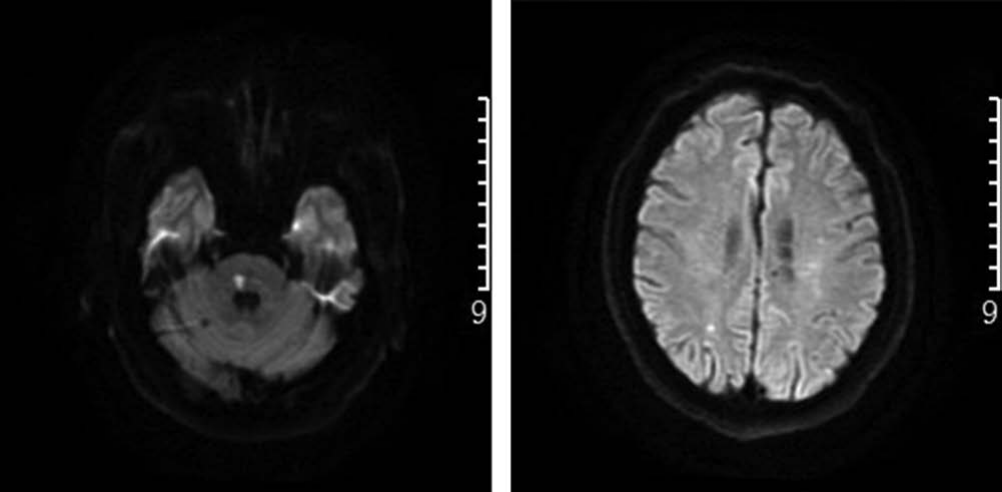
Brain MRI DWI (June 2021): right pontin and right parietal lobe acute infarct lesion. DWI = diffusion-weighted imaging, MRI = magnetic resonance imaging.

**Figure 2.**
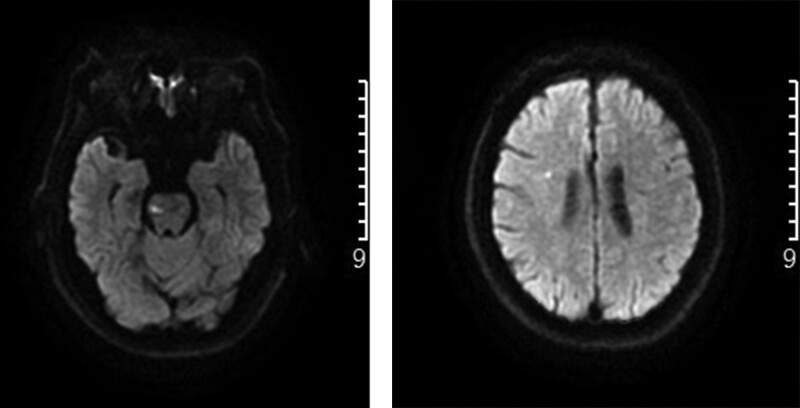
Brain MRI DWI (December 2021): right pontin and right frontal lobe acute infarct lesion. DWI = diffusion-weighted imaging, MRI = magnetic resonance imaging.

**Figure 3.**
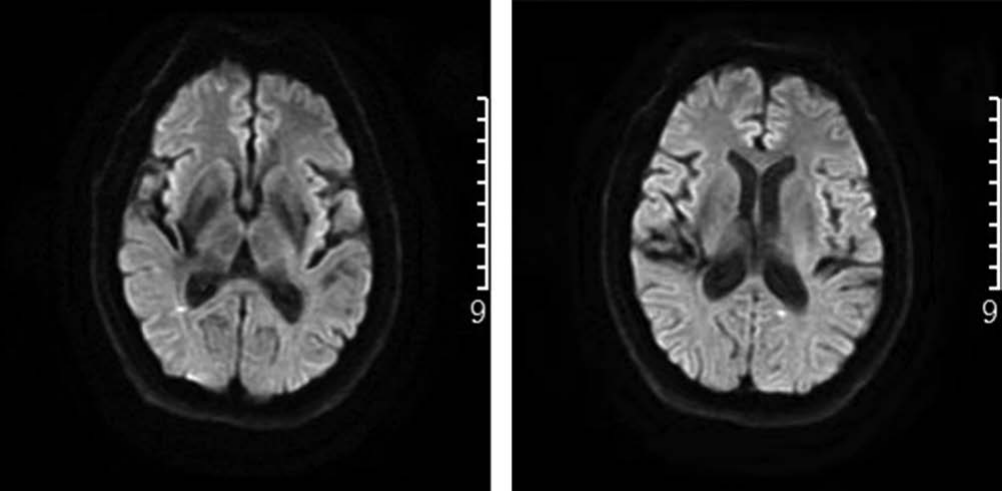
Brain MRI DWI (January 2022): acute infarction lesion near the posterior horn of bilateral lateral ventricles. DWI = diffusion-weighted imaging, MRI = magnetic resonance imaging.

*Present illness*: The patient had 5 ministrokes within 14 months: November 2020, June, September, and December 2021, and January 2022. Each time, the patient presented left limb weakness, with or without slurred speech. The National Institutes of Health Stroke Scale: 2 to 5. Several small new infarcts were detected on brain magnetic resonance imaging (MRI) and diffusion-weighted imaging. Typical lesions are shown in Figures 1–3. No stroke-related abnormality was detected in multiple TTE and dynamic electrocardiography examinations, and no stroke-related artery disease was observed in head and neck computerized tomography angiography.

*Diagnosis*: Multiple cerebral infarctions (small artery occlusion). Aspirin and clopidogrel were administered to patients as dual antiplatelet therapy after the previous 4 attacks. Then, the dual antiplatelet was changed to single drug antiplatelet therapy with clopidogrel after 3 weeks, accompanied by statins and antihypertensive and hypoglycemic therapy. Subsequently, the symptom of stroke improved but recurred after several weeks or months.

*History*: hypertension for >10 years, diabetes mellitus for 7 years, regular medication, blood pressure control at 130–150/90–100 mm Hg, and no blood glucose monitoring.

*Personal history*: Smoking for 30 years, >40 cigarettes/d.

*Family history*: The patient’s parents died of unknown etiology. He had no brother or sister, and his children were healthy.

*Physical examination*: Body temperature: 36.4 °C, pulse: 78 times/min, respiration: 18 times/min, blood pressure: 134/88 mm Hg, a clear mind, mild dysarthria, equal size and circles of bilateral pupils, with diameter 3.0 mm, sensitive to light reflection. No abnormal movement of bilateral eyeballs was detected in any direction, and no nystagmus was observed. Bilateral forehead wrinkles were symmetrical, the left nasolabial sulcus was slightly shallow, the tongue stuck out to the left, and the bilateral pharyngeal reflex was normal. The muscle strength of the limbs was grade 5, and the muscle tone was moderate. No abnormality was detected in the deep and shallow sensory examinations. Finger/nose, heel-knee-shin tests were normal, Romberg’s sign: negative. Babinski’s sign: left side positive, right side negative. NIHSS score: 2 points; Water swallow test: level 2.

*Laboratory examination*: Routine blood test, coagulation function, liver and kidney function, electrolyte and blood lipid were normal, fasting blood glucose: 6.7 mmol/L, glycosylated hemoglobin: 6.5%.

*Dynamic electrocardiogram*: The basal heart rate was sinus rhythm with an average heart rate of 74 beats/min. No persistent or paroxysmal atrial fibrillation (or atrial flutter) was recorded.

**Figure 4.**
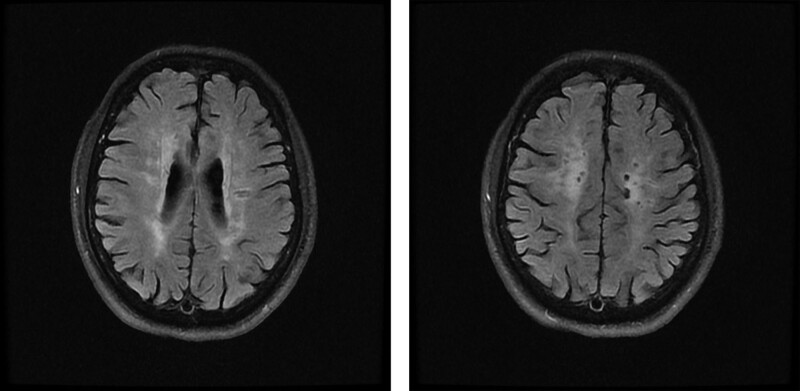
Brain MRI FLAIR: white matter lesions. FLAIR = fluid-attenuated inversion recovery, MRI = magnetic resonance imaging.

*Imaging examination*: Brain MRI T1 weighted imaging, T2 weighted imaging, and fluid-attenuated inversion recovery identified White matter lesions as shown in Figure 4.

**Figure 5.**
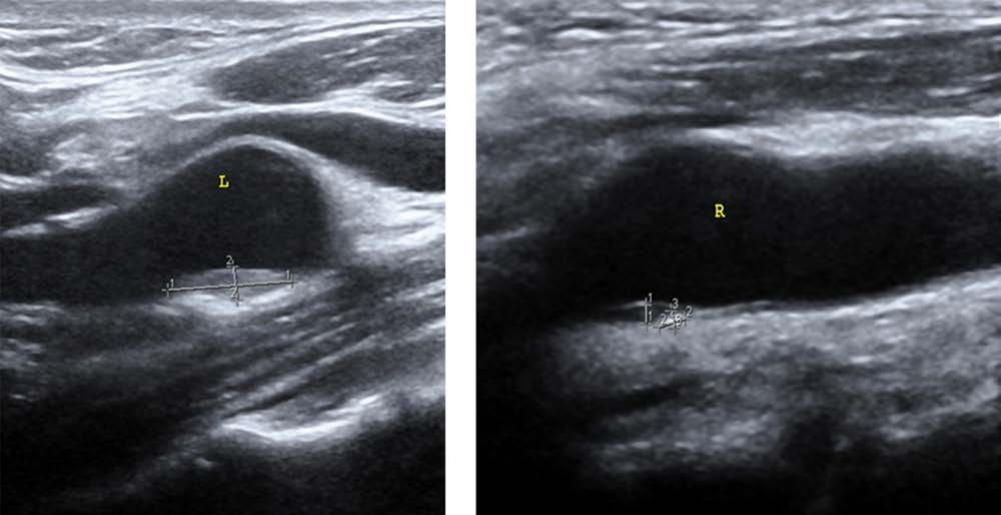
Carotid artery ultrasound: plaque formation at the left common carotid artery bifurcation and right internal carotid artery origin.

*Carotid artery ultrasound*: Plaques formed at the bifurcation of the left common carotid artery and the origin of the right internal carotid artery. Moreover, no abnormal echo was detected in the bilateral vertebral arteries, as shown in Figure 5.

**Figure 6.**
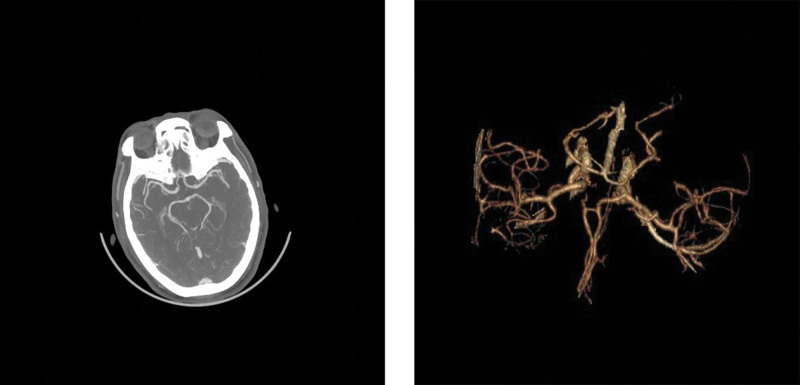
Head and neck CTA: slight stenosis of siphon segment of bilateral internal carotid arteries and slender right anterior cerebral artery. CTA = computerized tomography angiography.

*Head and neck computerized tomography angiography*: The right anterior cerebral artery A1 segment was thinner than that of the left side, and calcium plaque formed in the aortic arch and siphon segment of bilateral internal carotid arteries with slight stenosis of corresponding lumens, as shown in Figure 6.

**Figure 7.**
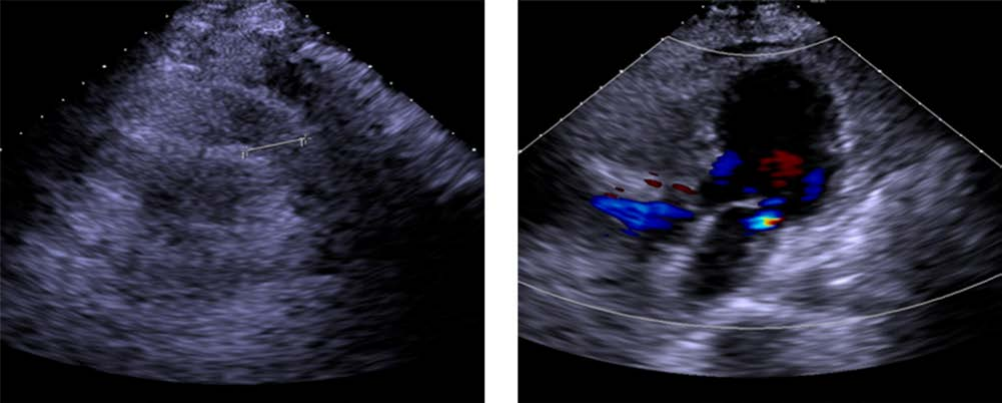
TTE: aortic valve degeneration and tricuspid insufficiency. TTE = transthoracic echocardiography.

*Transthoracic echocardiography*: Aortic valve degeneration and tricuspid insufficiency is observed (Fig. 7).

**Figure 8.**
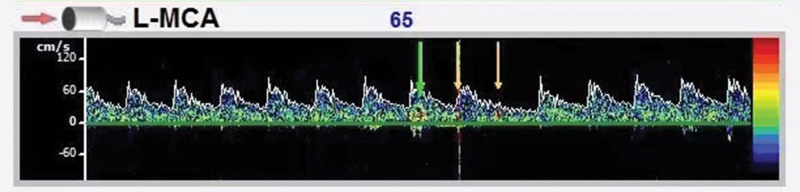
c-TCD: after 10 seconds of rapid intravenous injection of 10 mL activated normal saline, 3 to 4 microembolic signals were observed in the left middle cerebral artery under calm breathing state. The patient was unable to cooperate with the Valsalva action. c-TCD = contrast-enhanced transcranial Doppler.

*Contrast-enhanced transcranial Doppler*: Right-to-left shunt (grade 1, intrinsic type), as shown in Figure 8.

**Figure 9.**
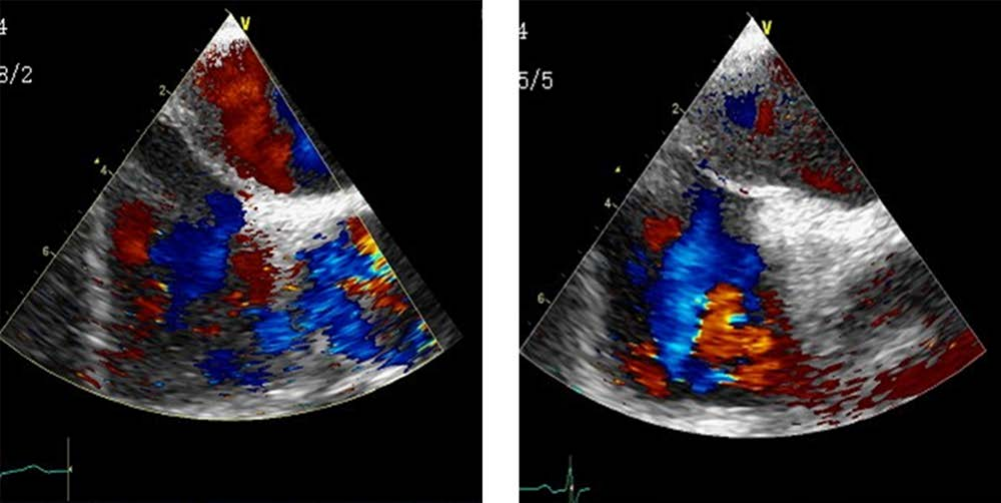
TEE: color ultrasonic flow between primary and secondary septum. The beam width of the ultrasonic color flow was about 0.14 cm, left-to-right shunt. TTE = transthoracic echocardiography.

TEE presented PFO, as shown in Figure 9.

**Figure 10.**
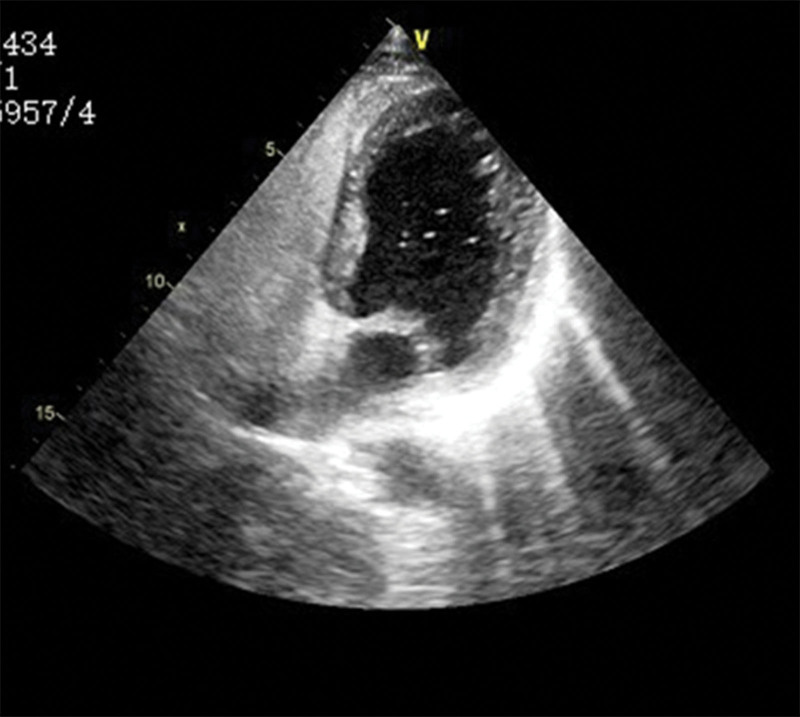
c-TEE: the temporarily prepared ultrasonographic contrast agent was rapidly injected through the elbow vein of the left upper limb to observe the sequence of atrioventricular development in the heart. After the development of the right atrium and ventricle, bubbles could be seen entering the left atrium, left ventricle, and ascending aorta. The patient could not cooperate with Valsalva action. The left cardiac system was observed after the contrast medium was administered twice. c-TEE = contrast transesophageal echocardiography.

Contrast transesophageal echocardiography (c-TEE) showed PFO, as shown in Figure 10.

*Diagnosis*: Cerebral infarction (cardiogenic), PFO.

*Treatment*: Based on the cardiovascular surgery consultation and combined with the wishes of the patient, the closure of PFO was suspended. An equivalent of 20 mg qd Rivaroxaban was administered orally to the patient.

*Prognosis*: No stroke occurred since the adjustment of the treatment plan.

*Feeling of the patient*: The patient was satisfied with the new treatment without adverse reactions.

## 3. Pathological findings

The patient did not undergo pathological examination.

## 4. Discussion

This study reported a clinical case of recurrent ischemic stroke in a short period, which was misdiagnosed as cerebral small vessel disease several times and finally diagnosed as PFO-AS by c-TCD, TEE, and c-TEE. The diagnosis process was circuitous and representative and needed further exploration.

PFO is common in adults, with a detection rate of about 25%.^[6]^ Previous studies have shown that the detection rate of PFO in patients with embolic strokes of undetermined source is about 40%.^[4]^ However, the presence of other risk factors cannot be used as evidence to exclude the pathogenesis of PFO. Huber et al^[7]^ demonstrated that after adjusting for age factors, for patients suffering from cryptogenic stroke without vascular risk factors, such as hypertension and diabetes mellitus, the detection rate of PFO was 36%, while the detection rate of PFO in patients with vascular risk factors was 20%. The study speculated that PFO-AS was likely to affect posterior circulation, which might be related to the higher blood flow velocity of posterior circulation than that of anterior circulation during Valsalva action.^[8]^ PFO-AS brain MRI lesions have the following characteristics: multiple, small, and scattered lesions are located in the vertebrobasilar artery region.^[9]^ Several studies suggested that PFO-AS patients who met the indications of transcatheter closure of PFO operation could benefit after the closure, and the other patients could benefit from the anticoagulant therapy.^[10]^ Additional studies have shown that for patients >60-years-old, the new oral anticoagulants have a better prevention effect on cryptogenic stroke than aspirin.^[11]^

This case was a 60-year-old male patient. He was a high-risk stroke patient due to the coexistence of hypertension, diabetes, smoking, and other vascular risk factors. The small, recurring, and scattered infarct lesions were detected mainly in the vertebrobasilar artery region. The secondary prevention of the antiplatelet was ineffective, and the pathogenicity of PFO was clear after combination with the results of c-TCD, TEE, and c-TEE. Although TTE was performed several times, PFO was not detected. Due to the coexistence of multiple vascular risk factors, the exclusion of atrial fibrillation, other common cardiogenic embolic sources (persistent atrial flutter, intracardial thrombus, artificial heart valve, atrial myxoma or other cardiac tumor, mitral stenosis, myocardial infarction within 4 weeks, heart failure with left ventricular ejection fraction <30%, valve excrescence, and infective endocarditis), intracranial and extracranial vascular stenosis, and other diseases (infectious diseases, hematological diseases, and vasculitis) based on relevant examinations, clinicians repeatedly misdiagnosed it as a cerebral arteriolar disease and ignored the further investigation of PFO. Due to the influence of several factors, such as body shape and pulmonary air, the detection rate of TTE for PFO was only 50–60%.^[1]^ TEE combined with c-TEE was the preferred method and gold standard for the diagnosis of PFO.^[2]^ However, TEE was traumatic and had the risk of arrhythmia, gastrointestinal bleeding, and airway spasm. The examination process was painful, with a low acceptance rate of patients, rendering it difficult for clinical application. Some studies have shown that c-TCD, as an indirect screening method for PFO, had a sensitivity of 96.8%, a specificity of 78.4%, and a consistency of 90% along with TEE.^[12]^ It is also noninvasive, cost-effective, simple, and easily accepted by patients. In conclusion, PFO is a common disease, and its pathogenic effect on stroke cannot be ignored because of other risk factors. TTE has a low detection rate of PFO, such that it is easily missed by TTE. On the other hand, c-TCD is easy to operate and can be popularized as a supplementary means for stroke etiological investigation and primary PFO screening.

## Author contributions

**Conceptualization:** Yiwen Zhang.

**Data curation:** Ruihan Zhu, Zhenzhen Yu, Songqi Hou, Jianping Niu.

**Formal analysis:** Ruihan Zhu.

**Methodology:** Yiwen Zhang, Songqi Hou.

**Resources:** Zhenzhen Yu.

**Validation:** Jianping Niu.

**Writing – original draft:** Yiwen Zhang.

**Writing – review & editing:** Yiwen Zhang.
